# Type of treatment, operated organ and sexual functioning of patients with cervical cancer

**DOI:** 10.1007/s00520-025-10101-y

**Published:** 2025-11-01

**Authors:** Krzysztof Manterys, Magdalena Błażek, Anna Kowalczyk

**Affiliations:** 1 Division of Quality of Life Research, Medical University of Gdańsk, Gdańsk, Poland; 2 Department of Oncology and Radiotherapy, Medical University of Gdańsk, Gdańsk, Poland

**Keywords:** Cervical cancer, Sexuality, Cancer treatment, Quality of life

## Abstract

Cancer treatment affects every area of a patient's life. Currently available cancer treatment methods not only increase the chances of survival, but also challenge patients to return to their pre-disease lifestyle. This means that a patient's functioning both during and after the disease consists not only of satisfaction in the spheres of family life and work life, but also sexual functioning. The primary objective of the presented study was to assess the sexual functioning of female patients, taking the type of treatment, quality of life domains and sociodemographic factors into account. The study was conducted on 70 women between the ages of 32 and 84 at the Department of Oncology and Radiotherapy of the University Clinical Center, and the Department of Obstetrics and Gynecology, Gynecologic Oncology and Gynecologic Endocrinology in Gdansk, Poland in 2021. The results indicate that patients who experience less discomfort and have better ratings of their overall functioning also report better ratings of their sexual functioning, and are less likely to report irritating complaints. Women treated with radiation therapy report more difficulties in functioning than patients who did not receive this treatment.

**Supplementary Information:**

The online version contains supplementary material available at 10.1007/s00520-025-10101-y.

## Background

Cervical cancer ranks as the fourth most common cancer among women in terms of incidence. Data show that about one million women are diagnosed this type of gynecological cancer every year, of which about 300,000 cases result in patient death [1].

As a result of the disease and the changes in their bodies, patients with cervical cancer may experience a crisis of femininity, lowered self-esteem and decreased self-worth [2–5]. A decrease in libido and sexual satisfaction can be observed in the area of sexual functioning [2, 4, 6, 7]. In addition, libido and feelings of negative mood are affected by persistent treatment-related pain, as well as chronic fatigue related to the duration of the disease and cancer therapy [6].

When speaking about cervical cancer treatment, mention is usually made of hormone therapy, radiation therapy, radio-chemotherapy and/or surgery (such as radical hysterectomy). Importantly, regardless of the treatment strategy chosen, the researchers note that patients may suffer from symptoms such as vaginal dryness, irritation and atrophy of the vulva and vagina, as a result of their cancer treatment. Regardless of the treatment method, all forms of cancer therapy can cause: premature menopause, vaginal narrowing and decreased vaginal elasticity, loss of vaginal sensation, urinary incontinence, diarrhea, rectal pain and cystitis [8].

The type of treatment used may also differentiate patient groups in terms of their assessment of sexual functioning. Radiation therapy treatment leads to functional and anatomical changes in the patient's vagina, and can result in characteristic difficulties and discomforts in terms of both physical (e.g., lymphedema, diarrhea, constipation) and psychosocial areas (e.g.,lack of desire to interact with people, reduced mood) [9, 10]. Despite its proven effectiveness in treating cervical cancer, radiation also interferes with the psychosexual sphere of women, resulting in symptoms of vaginal dryness, lack of vaginal lubrication, short inflexible vagina, premature menopause, infertility, dyspareunia, pain during sexual intercourse, and lack of sexual satisfaction [11–13]. A study by Diniz et al. (2020) compared two patient groups: those treated with radiation therapy, and those not treated with radiation. The authors observed that patients with cervical cancer reported difficulties related to sexual desire and arousal significantly more often after treatment with radiation therapy than patients with no radiation therapy [14]. The effects of cervical cancer surgery are invisible, or at least easy to mask in daily life. Studies show that female patients treated with surgery assess their quality of life well in the social and professional aspects, but not in the psychosexual aspect. This is because the disease and the surgery can affect the patient's self-esteem, perception of her body image, and ability to feel pleasure during sexual intercourse.

Sexuality is one of the important determinants of health-related quality of life [2–7, 9, 15]. In oncology, sexuality is a particularly sensitive area due to the fact that both the disease- and the treatment-related side effects impair the desire and ability to be sexually active, resulting in patients’ deteriorating overall well-being and functioning. Sexual dysfunction is primarily due to the inability to satisfy basic needs, and the causes of sexual dysfunction from cervical cancer are multidimensional, and are not only due to the disease symptoms and the treatment method, but also occupational situation, social situation, socio-demographic conditions, support from loved ones and, specifically important: acceptance and affection from the partner. Symptoms of sexual dysfunction are reported more often by women who are older, those from small towns and those with lower education. Socially, this presents particular importance, as lower education levels may be associated with lower self-awareness concerning diseases and preventive health care, while coming from smaller towns may limit access to medical assistance and health promotion [8]. In addition, cervical cancer can also affect one’s relationship with partner/husband. This means that an essential part of the relationship is impaired, potentially leading to additional concerns about the relationship itself. According to patients’ estimation, the better her sex life was before the disease, the worse her well-being may be during the disease [3, 5, 16, 17].

The main aim of the study was to analyze the relationship between:

1. Sexual functioning and quality of life assessment and age
2. And also to compare women depending on the type of treatment chosen in terms of quality of life assessment and sexual functioning. It was assumed that:
  1. The assessment related to coping with the disease will be related to sexual functioning and quality of life
  2. Age will correlate with the response to treatment and sexual functioning. The study also presented several socio-demographic variables, including: age, education and place of residence of the surveyed patients.

## Methods

### Study participants

The study involved 70 patients with cervical cancer treated at the University Clinical Center in Gdansk. Among the surveyed women with cervical cancer, the largest group included patients with secondary education (n = 37). The patients ranged in age from 32 to 84 years, with a mean age of M = 53.71 (SD = 12.50).

Respondents qualified for the study were diverse in terms of education.

Among cervical cancer patients, the largest number of respondents had secondary education – 52.86%. The second largest group included women with higher education – 20.00%, followed by patients with vocational education – 17.14%, while the smallest group consisted of women with primary education – 10%.

Among the patients, the largest proportion of the study participants lived in a city with a population of 25,000 to 100,000 residents – 35.72%. The second largest group included women living in cities with more than 100,000 residents – 34.29%, followed by villages – 28.57%, while the smallest group consisted of those living in cities with up to 25,000 residents – 1.43%.

In our study, most of the patients were married – 51.43%. Patients who were not in a relationship were the second most numerous group, constituting 24.29%, followed by women in informal relationships, at 17.14%. The other female participants did not provide information about having a partner – 7.14%.

Patients were also studied in terms of medical procedures variations used in the course of treatment.

Among women with cervical cancer, over 87% of the patients underwent surgery and were on average 9 years (me = 2016) after the surgery. The remaining patients (13%) were waiting for surgery.

Radiotherapy was used in 38.57% of patients and was on average 8 years after treatment with this method (me = 2017). During the study, none of the patients were undergoing radiotherapy.

Chemotherapy was used in 31.43% of the study participants, while 7.14% received hormone therapy.

Among the patients, 32.86% had previously received combined treatment (e.g., radiation + chemotherapy).

### Methods

The following methods were used in the study:

1. The EORTC QLQ C30 (version 3.0) was created by the European Organization for Research and Treatment of Cancer (EORTC) [32]. This is a questionnaire used to assess the quality of life in cancer patients. It includes four-item scales for the first five questionnaire items, describing physical functioning (PF;1–5), social role functioning (RF;6,7), emotional functioning (EF;21–24), memory and concentration (CF;20,25), social functioning, and three scales describing disease symptoms (fatigue (FA;10,12,18), nausea and vomiting (NV;14,15), and pain (PA;9,19)). The responses cover four categories – not at all, a little bit, a lot, and very much. To accommodate these categories, question 4 was reworded as "Do you need help eating, dressing, washing yourself or using the toilet?". The last two items (QL;29,30) refer to the overall assessment of health status and quality of life. They were presented on a seven-point scale, where 1 means "very bad" and 6 means "excellent."
2. The EORTC-CX24 questionnaire is a 24-item tool specifically designed to assess the quality of life of women with cervical cancer, and includes symptoms related to the disease itself, as well as the course of treatment. Patients answer questions specifying the severity of the analyzed parameters on a 4-point scale ("never" (1), "sometimes" (2), "often" (3), "very often" (4)). The tool consists of 3 multi-item and 6 single-item subscales: SV (Sexual/Vaginal Functioning Scale); BI (Body Image Scale; items 45–47); SE (Symptom Experience Scale; items 50–53); LY (Lymphoedema; item 38); PN (Peripheral Neuropathy; item 40); MS (Menopausal Symptoms; item 44); SXW (Sexual Worry; item 48); SXA (Sexual Activity; item 49); SXE (Sexual Enjoyment; item 54).

All the scales in question have been recoded to a range of 0–100 points, so that a higher value obtained on the scale indicates greater severity of the studied trait.

3) Assessment of female sexual functioning (Female Sexual Function Index). This is a questionnaire used to assess all aspects of a woman's sexual functioning. It includes two 5-point scales for the first two questionnaire items describing sexual desire and five 6-point scales describing: arousal, lubrication, orgasm, sexual satisfaction and sexual pain complaints. The first scale was presented on a five-point scale, where 1 means "Almost never or never," and 5 means "Almost always or always." For the other five scales, the scores were described from 0, which means "I have not had sexual intercourse," to 6, which means "Almost never or never."

The application to conduct the study was verified and accepted by the Independent Bioethics Committee of the Medical University of Gdańsk (statute no. NKBBN/679–595/2020). Consent to the study operates in accordance with the regulation of the Minister of Health in Poland and the Helsinki Declaration. Consent to the study was obtained in writing from patients. During the COVID-19 quarantine periods, consent to the study was obtained by talking to a nurse and signing a form. In cases where there was no risk to the patient's health, the proposal to participate in the study, along with the potential signature, was conducted in person at the clinic by the researchers.

## Results

Statistical analyses were performed using Statistica 13 (Poland, Gdansk) using correlation analysis and comparison of mean measures using the parametric Student's t-test, Shapiro–Wilk test, Levene's test and Mann–Whitney U test.

### Relationship between domains of sexual functioning and quality of life and age in a group of women with cervical cancer

A moderate negative correlation was observed between age and the symptom scale of the EORTC-CX24 questionnaire (r = −0.42, *p* = 0.00), but due to the inverse nature of the questionnaire's scoring, this result actually represents a positive correlation (the older the patient, the more complaints she reported on the symptom scale) (Table 1).

**Table 1:** Relationship between age and quality of life assessment

Age	R	p-value
EORTC-cx-24 Symptom Scales	-.42	0.000
EORTC-cx-24 Functional Scales	-.32	0.008

Low negative correlations were observed between age and the functioning scales of the EORTC 30 questionnaire (r = −0.39, *p* = 0.001) and the EORTC-24 questionnaire (r = −0.32, *p* = 0.08) (the older the patient, the worse she rated her functioning on both questionnaires) (Table 1).

Several negative correlations were detected between age and the scales of arousal (r = −0.36, *p* = 0.002), lubrication (r = −0.28, *p* = 0.019) and overall sexual functioning (r = −0.24, *p* = 0.047) (the older the patient, the worse she rated her functioning in the areas of arousal, lubrication and overall sexual functioning).

### Relationship between domains of quality of life in a group of women with cervical cancer

A strong positive correlation was detected between the functional scale of the EORTC-30 questionnaire and the EORTC-30 symptom scale (r = 0.79, *p* = 0.00), but due to the inverse scoring of the symptom scale, the result actually indicates an inverse correlation (the better the functional score, the fewer complaints reported). Similar results were observed between the EORTC-30 functioning scale and the EORTC-CX-24 symptom scale (r = 0.75, *p* = 0.00) (Table 2).

Both symptom scales were found to be positively correlated (r = 0.76, *p* = 0.00), meaning that the fewer symptoms patients reported on the EORTC-30 scale, the fewer symptoms they reported on the EORTC-CX-24 scale (due to the inverse nature of the symptom scale, the score actually indicates an inverse correlation). The results are presented in Table 2.

**Table 2:** Relationship between domains of quality of life in a group of women with cervical cancer

	EORTC-30 Global Health status	EORTC-30 Functional Scales	EORTC-30 Symptom Scales	EORTC-cx-24 Symptom Scales	EORTC-cx-24 Functional Scales
EORTC-30 Functional Scales	-.47 *p* =.000	1.00 *p* = ---	.79 *p* =.000	.75 *p* =.000	.27 *p* =.022
EORTC-30 Symptom Scales	-.44 *p* =.000	.79 *p* =.000	1.00 *p* = ---	.76 *p* =.000	.29 *p* =.015

### The relationship between domains of sexual functioning

A low positive correlation was detected between the desire scale and the arousal scale of the FSFI questionnaire (r = 0.25, *p* = 0.041) (the higher the patient rated her feelings of sexual desire, the better she rated her ability to be aroused).

Moderate to strong positive correlations were observed between the sexual arousal scale and the scales of lubrication (r = 0.88, *p* = 0.000), orgasm (r = 0.87, 0.000), sexual satisfaction (r = 0.57, 0.000) and overall sexual functioning (r = 0.91, *p* = 0.000).

Similar results, in terms of type and level of correlation, were obtained between the lubrication scale and the scales of orgasm (r = 0.93, *p* = 0.000), sexual satisfaction (r = 0.62, *p* = 0.000) and overall sexual functioning (r = 0.94, *p* = 0.000).

The orgasm scale also showed positive correlations. In our study, the orgasm scale correlated strongly with the scales of lubrication (r = 0.93, *p* = 0.000), sexual satisfaction (r = 0.72, *p* = 0.000) and overall sexual functioning (r = 0.97, *p* = 0.000).

High positive correlations were detected between the pain complaints scale and scales of: arousal (r = 0.71, *p* = 0.000), lubrication (r = 0.73, *p* = 0.000), orgasm (r = 0.79, *p* = 0.000), sexual satisfaction (r = 0.65, *p* = 0.000) and overall sexual functioning (r = 0.85, *p* = 0.000). Given the inverse nature of the pain scale, the correlations presented show that the less pain patients experience, the better they rate their sexual functioning.

In addition, a strong positive correlation was detected between the general sexual functioning scale and the sexual satisfaction scale (r = 0.77, *p* = 0.000).

### Relationship between domains of sexual functioning and quality of life in a group of women with cervical cancer

A low positive correlation was observed between the desire scale of the FSFI questionnaire and the global quality of life rating of the EORTC-30 questionnaire (r = 0.29, *p* = 0.014) (the better the patient rated her sexual desire, the better her global quality of life rating).

Interestingly, the study shows average and strong positive correlations between the EORTC-24 questionnaire functioning scale and the FSFI questionnaire scales: arousal (r = 0.73, *p* = 0.000), lubrication (r = 0.72, *p* = 0.000), orgasm (r = 0.72, *p* = 0.000), sexual satisfaction (r = 0.52), and overall sexual functioning (r = 0.74, *p* = 0.000) (the better the patient rated her quality of life, the better she rated domains in the sexual sphere).

### Comparison of averages for two independent groups (women who underwent radiation therapy vs. patients who did not, chemotherapy vs. patients who did not, hormone therapy vs. patients who did not, surgery vs. patients who did not undergo surgery) in the areas of quality of life and sense of control questionnaire (*p* = 0.03

T-test was performed for two groups of patients, taking quality of life assessment and sense of control into account.

A categorized histogram was generated for each variable, followed by the Shapiro–Wilk test. None of the variables was higher than 0.05, which did not allow us to reject the null hypothesis and perform the Student T-test.

Since all variables did not meet the normality of distribution condition, we performed a non-parametric test (in the Shapiro–Wilk test for the remaining variables, p was lower than 0.05).

The Mann-Whiney U test confirms that the differences in medians observed only between the group of women treated with radiation therapy (and the group of patients who did not receive this treatment) are statistically significant. The results indicate that the variable is statistically different for the two groups of female subjects only in the complaints scale of the EORTC-30 questionnaire (*p* = 0.03) (Table 3).

**Table 3:** Mann–Whitney U test (with correction for continuity)

	Rank.sum Untreated	Rank.sum Treated	U	Z	p-value	N valid. Untreated	N valid. Treated women
EORTC 30 - SYMPTOM SCALES	1347.5	1137.5	401.500	−2.15904	0.031	43	27

Individual combined and single treatment methods (e.g. radio-chemotherapy versus radiotherapy alone) were also compared and no significant results were obtained in these areas.

## Discussion

Of all cancers in the female cancer population, gynecological cancers present the greatest negative effect on sexual functioning and perceived body image [2].

In our survey, we primarily assessed sexual quality of life. Our results indicate that the higher a patient's rating of sexual desire ability, the better her rating of arousal ability. Sexual arousal also correlated strongly positively with the ability to achieve lubrication, orgasm, sexual satisfaction and overall sexual functioning, demonstrating that the FSFI questionnaire scales are reliable and consistent. We obtained similar positive results for the orgasm scale.

Importantly, pain complaints were also found to be significantly correlated with scales of lubrication, orgasm, sexual satisfaction and overall sexual functioning, meaning that the greater the pain the patients experienced during sexual contact, the worse they rated their sexual functioning. This result is consistent with the results of other authors, who indicate that pain caused by cancer treatment can lead to cessation of sexual activity and sexual dysfunction [8, 11, 12].

Since sexuality is closely linked to health-related quality of life, we also looked at how quality of life can be linked to sexual activity. Our results indicate that the better the patient rated her sexual functioning, the better she rated her quality of life, which is consistent with the findings of other authors [5, 14, 17].

We also paid attention to sociodemographic variables. The results indicate that the older the patient was, the more complaints she reported on the symptom scale. This is somehow related, as the more symptoms the patient reported, the worse she rated her overall functioning and quality of life. Interestingly, both symptom scales appeared to be consistent, and if patients reported complaints on one scale (EORTC-30), they reported similarly on the other (EORTC-24). At the same time, the group of cancer patients does not seem to differ from the group of healthy women. With age, the number of reported complaints increases, and in our study we observed this in patients with CC.

Our results showed that age was also related to the assessment of overall functioning in the disease, wherein the older the patient was, the worse she assessed her functioning. What is important, the older the patient was, the worse she rated her ability to achieve arousal, lubrication and overall sexual functioning. It seems understandable that sexual function can diminish with the process of aging. This is in line with the findings of other researchers who noted that the correlation of age with cervical cancer leads to a worsening of the overall assessment [8].

In our study, we also looked at how the treatment type can affect patient functioning. According to the results presented above, the patients who were treated with radiotherapy reported significantly more complaints than those who did not receive such treatment. Many authors point out that radiation therapy is an invasive treatment that, in addition to its effectiveness in treating cancer, can also cause many side effects, including those related to sexual functioning [14, 16].

According to numerous studies, between 90 and 100% of cervical cancer patients face sexual dysfunction related to symptoms of vaginal dryness, dyspareunia and low libido. Sexual dysfunction is also associated with psychological symptoms resulting from the effects of the disease manifesting in every area of life. As a result of the disease, patients may feel anxiety, symptoms of depression, stress and isolate themselves from society and loved ones [2, 5–7].

In our study, we looked at how the treatment type modulates women's sexual behavior and potentially affects quality of life scores.

The lack of a sufficient female subject number may have affected the quality and accuracy of correlative studies. The fact that women with cervical cancer account for about 2% of cancer patient population still does not explain the number of participants in the study.

In one study, as many as 100% of cervical cancer patients faced sexual dysfunctions as a result of the disease and the treatment process, which is consistent with the results of our study and other authors of research in this area and motivates in-depth patient follow-up with regard to sexual dysfunctions [2–7].

The main limitation of this study is, of course, the small group size of surveyed women. As COVID-19 restrictions prevailed for the study duration, the researchers faced restrictions on patient access. Another issue is that there are no centralized support groups directly dedicated to this female group in Poland, contrary to, e.g., breast cancer patients. An additional limitation of the study was the significant difference in age in the study group. In the future, it would be worth creating a study, dividing the age groups into sections, e.g. every ten years, to carefully observe potential differences in coping with treatment and sexual functioning depending on the developmental stage.

The whole world sees new recommendations and discoveries in the field of cancer treatment practically every year. This makes patients live longer, not have to experience physical pain and allows them to plan their future. At the same time, successful treatment process completion is not, or at least should not be, the end of support from doctors and other experts in oncology or related sciences. Having considered the foregoing, the field of oncology treatment research has undergone significant development and expansion. Working with cervical cancer patients has been under-represented in Poland before. There was a lack of information about what happens to these women during treatment, what their needs, concerns and worries are. Because of that, this type of research presents extraordinary value, because it allows us to pay attention to what challenges such a specific group of patients face.

The creation of a support group, a community for females with cervical cancer will not only facilitate access to patient contact, but most importantly educate, raise awareness and support affected women, so that the patients would increase their awareness and ask specialists in the field of psychology and sexuality for support.

The intention of the researchers, in this particular work, is to inspire facilities, both public and private, to introduce psychology, psychotherapy and sexology support, so that the cervical cancer patient group could be noticed, at least the same as breast cancer patients. It is worth creating hospital support groups or societies that gather women experiencing cervical cancer in order to gain mutual support and knowledge about coping with the disease and returning to life activity to the best of their abilities. Additionally, the study aimed to increase doctors' awareness of the scale and multidimensionality of the problem of cervical cancer.

## Supplementary Information

Below is the link to the electronic supplementary material.

ESM 1 (PDF 349 KB)

## Data Availability

No datasets were generated or analysed during the current study.
