# Supplementary material for: Type of treatment, operated organ and sexual functioning of patients with cervical cancer
Source: Support Care Cancer. 2025 Nov 1;33(11):1004. doi: 10.1007/s00520-025-10101-y (PMC12578740; doi:10.1007/s00520-025-10101-y)
Supplement: Supplementary file 1 — (PDF 349 KB) [file 520_2025_10101_MOESM1_ESM.pdf]

Formularz świadomej zgody pacjenta

Ankieta służy do oceny jakości życia pacjentek leczonych onkologicznie. Celem tej pracy jest pomiar poczucia wpływu na stan zdrowia i funkcjonowania seksualnego w trakcie leczenia u kobiet z rakiem piersi lub szyjki macicy.

Tytuł badania naukowego**: Umiejscowienie poczucia kontroli a jakość życia u pacjentek z nowotworem piersi i szyjki macicy.**

Numer pacjentki/pacjenta..

wiek..................płeć..............................

Zapoznałam/em się z przedstawioną mi informacją o badaniu naukowym. Treść jej zrozumiałam/em. Autor badania odpowiedział na wszelkie moje pytania.

Wyrażam świadomą zgodę na uczestnictwo w badaniu naukowym co obejmuje:

- wypełnienie kwestionariuszy

.... ………………………………………

Podpis pacjentki/pacjenta Podpis osoby odbierającej zgodę pacjenta

Miejscowość………………………data................................
